# Correction: Neurodevelopmental performance among pre-schoolers treated for severe anaemia at Lira Regional Referral Hospital, Uganda

**DOI:** 10.1371/journal.pone.0244913

**Published:** 2021-01-05

**Authors:** The PLOS ONE Staff

## Notice of republication

This article was republished on November 16, 2020, to correct an error in the title that was introduced during the typesetting process. The publisher apologizes for the errors. Please download this article again to view the correct version. The originally published, uncorrected article and the republished, corrected articles are provided here for reference.

## Supporting information

S1 File Originally published, uncorrected article. (PDF)

S2 File Republished, corrected article. (PDF)
