# Supplementary material for: Correction: Neurodevelopmental performance among pre-schoolers treated for severe anaemia at Lira Regional Referral Hospital, Uganda
Source: PLoS One. 2021 Jan 5;16(1):e0244913. doi: 10.1371/journal.pone.0244913 (PMC7785225; doi:10.1371/journal.pone.0244913)
Supplement: S2 File — (PDF) [file pone.0244913.s002.pdf]

PLOS ONE

RESEARCH ARTICLE

Neurodevelopmental performance among pre-schoolers treated for severe anaemia at Lira Regional Referral Hospital, Uganda

Andrew S. Ssemata[1]*, **Robert O. Opoka**[2], **John M. Ssenkusu**[3], **Noeline Nakasujja**[1], **Chandy C. John**[4], **Paul Bangirana**[1]

1 Department of Psychiatry, Makerere University College of Health Sciences, Kampala, Uganda, **2** Department of Paediatrics and Child Health, Makerere University College of Health Sciences, Kampala, Uganda, **3** Department of Epidemiology and Biostatistics, School of Public Health, Makerere University, Kampala, Uganda, **4** Ryan White Center for Pediatric Infectious Diseases & Global Health, School of Medicine, Indianapolis, Indiana, United States of America

* andrewssemata@yahoo.co.uk

Abstract

Background

Severe anaemia is a common clinical problem among young children in sub-Saharan Africa. However, the effect of severe anaemia on neurodevelopment of these children is not well described. Therefore, we assessed the neurodevelopmental performance of preschool children diagnosed with severe anaemia in Northern Uganda.

Methods

We conducted a prospective cohort study among children < 5 years of age 14 days post discharge after an episode of severe anaemia (Hb < 5.0 g/dl; n = 171; mean Hb = 3.9g/dl) at Lira Regional Referral Hospital, Uganda. Neurodevelopmental outcomes (cognitive, language and motor) were assessed using Bayley Scales of Infant and Toddler Development, 3rd edition (Bayley-III). Age-adjusted z-scores for each domain were calculated using scores from healthy community control children (n = 88) recruited from the same environment for each age category. Multiple linear regression was used to compare z-scores in the cognitive, language and motor scales between the two groups after adjusting for weight-for-age z-score, socioeconomic status, mother's education, and father's employment on all the scales.

Results

The prevalence of neurodevelopmental impairment was 2.3% (95% CI: 0.8–6.1) for cognition, 1.7% (95%: 0.6–5.3) for language and 3.5% (95% CI: 1.6–7.6) for motor scales and 4.6% (95% CI: 2.3–9.1) for deficits in ≥1 area of neurodevelopment. Significant differences were observed between the two groups with the SA group performing worse on cognition [adjusted mean score, (Standard error, SE), *P*-value] [-0.20, (0.01) vs. 0.00, (0.01), *P* = 0.02]; language [-0.25, (0.01) vs. 0.00, (0.01), *P*< 0.001]; and motor [-0.17, (0.01) vs. 0.00, (0.01), *P* = 0.05] scales.

OPEN ACCESS

Citation: Ssemata AS, Opoka RO, Ssenkusu JM, Nakasujja N, John CC, Bangirana P (2020) Neurodevelopmental performance among pre-schoolers treated for severe anaemia at Lira Regional Referral Hospital, Uganda. PLoS ONE 15(11): e0240694. https://doi.org/10.1371/journal.pone.0240694

Editor: Samson Gebremedhin, Addis Ababa University, ETHIOPIA

Received: April 21, 2020

Accepted: October 1, 2020

Published: November 4, 2020

Copyright: © 2020 Ssemata et al. This is an open access article distributed under the terms of the Creative Commons Attribution License, which permits unrestricted use, distribution, and reproduction in any medium, provided the original author and source are credited.

Data Availability Statement: All relevant data are within the manuscript. The dataset(s) supporting the conclusions of this article has been provided in the manuscript text and tables.

Funding: This work was supported by funding from grant number D43TW010132 from National Institutes of Health and a Fogarty International Centre grant number D43 NS078280, awarded to CCJ. The funders played no role in the study design, data collection and analysis, decision to

publish, or preparation of the manuscript. The contents of this work are solely the responsibility of the authors and do not necessarily represent the official views of the funders.

Competing interests: The authors have declared that no competing interests exist.

Conclusion

In children < 5 years of age, severe anaemia was associated with neurocognitive (cognition, language and motor) deficits in the immediate period post treatment. Further research is needed to identify risk factors and determine the long-term effects of poor neurodevelopment in young children with severe anaemia.

Introduction

Severe anaemia (SA) defined as haemoglobin (Hb) < 5g/dl is a global public health challenge commonly associated with childhood morbidity and mortality among children < 5 years of age in sub-Saharan Africa [1, 2]. Anaemia prevalence is estimated at 47.4% globally among preschool children with a burden of 62.3% among African children [3, 4]. In Uganda, the prevalence of anaemia among children < 5 years of age is estimated at 37.2% in the Northern part of Uganda [5]. The common causes of SA are multi-factorial including severe malaria, poor nutrition, micronutrient deficiencies (i.e. iron deficiency), sickle cell anaemia, acute illnesses and infections, but malaria is a major driver [6–8]. Despite several interventions to improve the iron status of children and reduce the anaemia burden, SA remains a challenge with variability in clinical management and community perceptions that affect seeking care and treatment [9, 10]. These interventions include blood transfusion [11, 12], iron supplementation/ giving haematinics [13, 14], de-worming [15], and malarial presumptive treatment [16, 17].

Severe anaemia in children can affect cerebral oxygen supply causing prolonged, repetitive and acute hypoxic-ischaemic events leading to cerebral damage, abnormalities and lesions in the basal ganglia, thalami, white and grey matter [18, 19]. These are significant risk factors for poor early childhood neurodevelopment. However, data on the association between SA and neurodevelopmental impairment is still limited. With improved survival rates and lowered mortality among children <5 years of age in our setting [10], we may potentially have more numbers of children surviving with neurodevelopmental impairment persisting long after treatment and resolution of anaemia.

Deficits in neurocognition in children may significantly impact future learning, class performance and academic attainment [20]. However, in routine clinical practice in the African setting, assessment of neurodevelopment in children surviving illnesses like SA is not integrated in routine clinical care [21, 22]. An earlier study that examined neurodevelopmental outcomes amongst Ugandan children < 5 years surviving severe malaria anaemia (SMA), a form of complicated malaria showed that SMA was associated with long-term cognitive impairment [23]. However, it remains unclear if the reductions in scores may be similarly observed for children surviving SA without malaria in the short-term post recovery. Given the association between anaemia and impaired cognition [24, 25], severe anaemia may also affect neurodevelopment significantly affecting full developmental growth. It is therefore essential to examine the association between exposure to SA and neurodevelopmental outcomes.

There is scanty literature to date on neurodevelopmental outcomes among children < 5 years of age with SA without malaria. Previous studies have not particularly focused on the much younger children in their first 1000 days of life, a time of critical brain growth, neural circuitry and development [26, 27]. Therefore, we aimed to estimate the prevalence of neurodevelopmental (cognition, language and motor) deficits among children below 5 years surviving SA at Lira Regional Referral Hospital in Northern Uganda where the prevalence of SA is 33.5% [28]. We hypothesized that children surviving SA will have poorer neurodevelopmental outcomes on all domains compared to healthy controls.

Materials and methods

This prospective cohort study was conducted at Lira regional referral hospital, Northern Uganda between August 2016 and June 2017. The hospital is a free-for-care hospital serving numerous districts in an area of all year high malaria transmission around the Lake Kyoga region [29]. Children are first seen in an outpatient section where those requiring admission are identified and taken to the paediatric ward as described elsewhere [10, 28].

Study participants

Children aged 6–42 months with SA (n = 180) with mean Hb = 3.9g/dl were consecutively enrolled from an ongoing implementation research study where SA was defined as Hb < 5g/dl [10, 28]. Children above 42 months and those with known sickle cell disease (a recognised specific cause of severe anaemia; equally associated with neurologic disability) [30] were excluded. All children with SA received a blood transfusion treatment at admission following the prevailing Ugandan Ministry of Health guidelines at the time of the study [31]. Classification, clinical guidelines and treatment management of these children is described elsewhere [10, 28]. Parents of children with SA were informed about the need for healthy control children of the same age from their nuclear or extended family (siblings) within the household compound area or neighbourhood (playmate) to generate a comparison/ control group. They were requested to bring the eligible children along with their caregiver (if different from child with SA) to the hospital when they returned for their assessment.

The healthy community controls (CCs) (n = 90) were of similar age (6–42 months) as children with SA and currently healthy. The CCs were recruited mainly as a normative group to generate z-scores and to serve as comparison with children with SA. The CCs were examined at the time of enrolment to ensure they were healthy and did not have: (1) clinical pallor on clinical examination; or (2) a history of hospitalization for severe anaemia 6 months or other illnesses 4 weeks prior to enrolment; or (3) major neurological, developmental or clinical abnormalities on physical examination. The participants were grouped to create appropriate and balanced 4 age bands (6–12 mo.; 12.1–24 mo.; 24.1–36 mo.; 36.1–42 mo.). The controls were recruited as a normative group to generate z-scores with at least 20 controls for each age band. The control group provided a comparison for the SA group and was intended to reduce norm related bias and provide a local normative reference score of the children using Bayley Scales of Infant and Toddler Development, Third Edition (Bayley–III) [32].

Sample size

Sample size was calculated done assuming a prevalence of cognitive deficits in Ugandan children at 26% [33]. A sample size of 180 children with severe anaemia was calculated to have 80% power and delta (decrease in prevalence) = 0.2, at 0.05 level of significance to demonstrate a two-fold increase in the risk of neurodevelopmental dysfunction in severe anaemia children. This sample size did allow for 4% non-responsiveness. Additionally, we recruited 90 controls with at least 20 controls for each of the 4 age bands as described above.

Demographic and clinical assessment

Demographic and socioeconomic status (SES) data were collected using a questionnaire checklist of material possessions previously used among the Ugandan paediatric population [34]. We assessed items involving nutritional status, child's education level, caregiver education level, housing quality in which lower SES scores have been associated with worse cognitive functioning in healthy Ugandan children < 5 years old [34]. SES questionnaire has a checklist

of indicators related to material possessions, house structure, living density, food resources and access to electricity, transport facility and clean water. Each indicator was given a score, and these scores are added to give a total SES score. A composite SES score using household's assets was determined and children categorized into wealth quintiles using principal components analysis [35, 36]. Height was obtained using a stadiometer with a sliding horizontal headpiece and weight using a paediatric calibrated weighing scale. Physical growth z-score anthropometric indicators (weight-for-age (WAZ), height for age (HAZ) and weight for height (WHZ) were used to evaluate nutritional status using WHO Anthro survey analyser software for children under 5 years of age; published norms and standardized z-scores. The WAZ, HAZ, and WHZ scores of less than −2 were categorized as underweight, stunting and wasting respectively [37, 38].

Neurodevelopmental assessment

The Bayley Scales of Infant and Toddler Development, 3rd Edition (Bayley–III) [39] were used for neurodevelopmental assessment at 14 days post discharge after clinical recovery for the children with SA and at enrolment for the community controls or when appropriate for the caregiver to bring the child to the hospital for assessment. All assessments were conducted in a quiet and child friendly room at the clinic and lasted approximately 50–70 minutes. The assessments were done in Langi, a local dialect as most children and caregivers were more fluent in Langi than English by competent psychology graduates trained in the child assessment and the use of Bayley-III. The two assessors were blinded to the child's study group. The first author (Health psychologist) supervised the administration of tests and reviewed the record forms at the end of each week for completeness to ensure data quality. The assessors were periodically given review trainings by a psychology graduate independent of the study and with expertise in Bayley-III. For consistency, the testers were video recorded conducting the assessments at the beginning-, mid- and end of the study and these were reviewed, discussed and feedback shared by the two testers and first author.

The Bayley-III is a widely used play-based standardized assessment measure of neurodevelopment in young children 1–42 months on various domains [39]. It evaluates a child in three areas: Cognitive (91 items), Language (97 items; subdivided into receptive—49 items and expressive communication—48 items subtests), and Motor 138 items; subdivided into Fine–66 items and Gross motor—72 items subtests). The Bayley-III tool used for neurodevelopmental assessment has not been validated for our setting however has been used in other studies and adapted for appropriate use among children in rural Uganda [40–42].

Statistical methods

Data were entered into Filemaker 11.0 v3 (FileMaker Inc. US) database, and exported into IBM SPSS 23 for statistical analysis. We compared the children with severe anaemia to healthy controls on the three neurodevelopmental summary scores (cognition, language and motor).

We converted raw scores for each scale into an age-specific standardized z-score, based on the scores of CCs since age has an effect on neurodevelopmental assessments where younger children will have lower raw scores than older children [33]. The z-scores were computed as (actual score–mean score for a child's sex and age)/SD, where the mean score for a child's sex and age and SD were computed by fitting a linear regression model to data for all CC children (n = 88) [23]. Z-scores have a mean of 0 and SD of 1 in the CC reference population. For all the scales, deficit was defined as a z-score of less than -2. Neurodevelopmental deficit was defined as deficit in more than 1 area (cognition, language and motor) as there was no way of generating an overall neurodevelopmental score from the primary data of scores in individual

areas. This has been used in previous cognitive studies among Ugandan children [33, 43]. Neurodevelopmental impairment prevalence for each domain was computed by dividing the number of children who had cognitive impairment in the SA group by the total number of children in the SA group.

Multiple linear regression was used to compare age- and sex-adjusted z-scores between the two groups while adjusting for weight-for-age z-score, socioeconomic status, mother's education, and father's employment (that differed between the two groups) for all the scales. We used the Holm–Bonferroni method (Holm procedure) to adjust for comparisons due to multiple testing [44].

Ethical considerations

Makerere University School of Medicine Research and Ethics Committee *REC-REF 2015–045*, Uganda National Council for Science and Technology *REF HS 2017* and Lira Hospital administration, approved the study. Caregivers of the study participants provided written informed consent.

Results

Characterization of the study children

One hundred eighty children with SA were recruited for the study. Of these, 06 did not return for assessment at day 14 post-discharge, leaving 174 children. During the assessment visit, 03 children had incomplete assessments, leaving 171 children who were analysed for neurodevelopmental performance. Additionally, 90 community control children were invited and consented to participate in the study. All the 90 children reported for their assessment however, two (02) children did not complete their assessments and 88 CCs were analysed for this study (Fig 1).

Of the 259 children (171 with SA and 88 CCs) aged 6–42 months with an overall mean age of 1.94 years (CCs, M = 2.07 years, SD = 0.96; SA, M = 1.88 years, SD = 0.94); 53% were boys

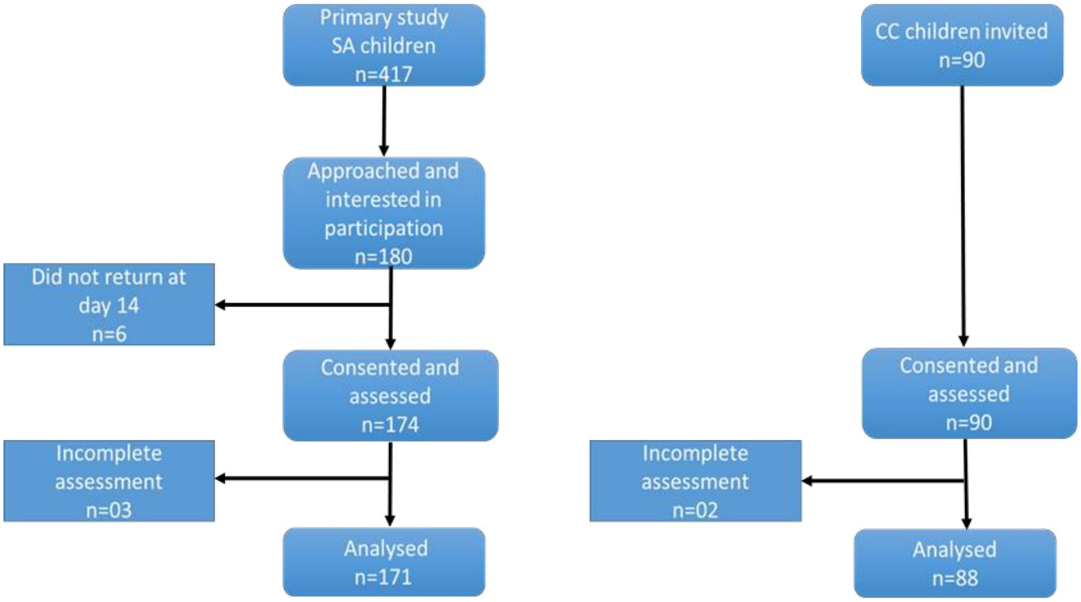

Fig 1. Study profile and assessment for the Bayley III.

https://doi.org/10.1371/journal.pone.0240694.g001

and 47% were girls. The pre-schoolers assessed in this study resided in the same geographical region. The socio-demographic characteristics of the study participants are represented in Table 1.

Neurodevelopmental performance

The present study assessed the prevalence and neurodevelopmental performance using age-adjusted z-scores of children with SA, compared to healthy CCs adjusting for socioeconomic status, WAZ, maternal education and paternal employment for all the main and sub-domains. Children with SA aged 6 to 42 months had a higher frequency of deficits in ≥1 area of neurodevelopment (8 of 171[4.7%]) (Table 2). The prevalence of neurodevelopmental impairment was 2.3% (95% CI: 0.8–6.1) for cognition, 1.7% (95%: 0.6–5.3) for language and 3.5% (95% CI: 1.6–7.6) for motor scales and 4.6% (95% CI: 2.3–9.1) for deficits in ≥1 area of neurodevelopment (Table 2).

With adjustment for multiple testing using Holm's procedure, significant differences were observed between the two groups with the SA group compared to CCs performing worse on cognition [adjusted mean score, (Standard error, SE), *P*-value] [-0.20, (0.01) vs. 0.00, (0.01), $P = 0.02$]; language [-0.25, (0.01) vs. 0.00, (0.01), $P < 0.001$]; and motor [-0.17, (0.01) vs. 0.00, (0.01), $P = 0.05$] scales (Table 3). Sub scale analysis showed significant differences in performance between children with SA compared to CCs for expressive communication [-0.32, (0.01) vs. 0.00, (0.12), $P<0.001$]; and gross motor [-0.25, (0.01) vs. 0.00, (0.01), $P = 0.02$] but not for receptive communication [-0.15, (0.01) vs. 0.00, (0.01), $P = 0.07$]; and fine motor [-0.08, (0.01) vs. 0.00, (0.02), $P = 0.29$] sub-scales (Table 3).

Discussion

The study assessed the prevalence of neurodevelopmental (cognition, language and motor) deficits among preschool children aged 6 to 42 months shortly after an episode of SA at Lira Regional Referral Hospital in Northern Uganda. The study found that children surviving SA had lower age unadjusted z-scores in the cognitive and language domains compared to CCs. After adjusting for SES, WAZ, maternal education and paternal employment and adjusting for multiple testing using Holm's procedure [44], children with SA had significantly lower scores in all the three domains (cognitive, language and motor).

In our study, SA was associated with small acute decreases in neurodevelopmental outcomes after multiple comparisons adjustment. The poor neurodevelopmental scores among children with SA compared to CCs may be subtle as we assessed the participants during recovery from severe anaemia. The study results reflect the potential effect of severe anaemia among pre-schoolers in resource-limited settings where the prevalence of SA among children is notably high [4]. Therefore, the current study provides evidence to date that SA is associated with immediate neurodevelopmental deficits in pre-schoolers in Northern Uganda. Hare [45] also observed that SA was associated with cerebral dysfunction and injury as the brain is vulnerable to anaemia-induced injury. Impaired cognitive function and memory among SA sufferers has been linked to a reduction of cerebral blood which affects neuronal function [25].

SA among children <5 years happens at a time of rapid child growth and development and increased iron requirements critical in proper brain growth with the potential to affect neurodevelopmental outcomes [46–48]. Children need iron to make haemoglobin, which carries oxygen to all body cells and iron deficiency has been associated with SA in pre-schoolers [49]. Therefore, the lack of iron during times of SA leads to decreased iron bioavailability and haemoglobin concentration to the central nervous system (CNS) thereby decreasing oxygen solubility of whole blood; inhibiting oxygen transportation [49]. This could lead to significant

Table 1. Demographic characteristics of severe anaemia and control study children.

Characteristic	Severe Anaemia (n = 171)	Community Children (n = 88)	P value
**Age in years, mean (SD)**	1.88 (0.94)	2.07 (0.96)	0.129
**Female sex, n (%)**	75 (43.9)	47 (53.4)	0.145
**Nutritional status indicators** [#]			
Underweight (WAZ<-2 SD) mean (SD)	10(6.2)	13 (14.9)	0.023*
Stunting (HAZ<-2 SD) mean (SD)	15(11.9)	10 (11.8)	0.975
Wasting (WHZ <-2 SD) mean (SD)	11(8.3)	12 (14.0)	0.284
**Socioeconomic status wealth indices, n (%)**			0.048*
Poor	44 (25.7)	10 (11.4)	
Second	30 (17.5)	21 (23.9)	
Middle class	31 (18.1)	24 (27.3)	
Fourth	36 (21.1)	20 (22.7)	
Wealthy	30 (17.5)	13 (14.8)	
**Maternal education level, n (%)**			0.015*
No school	12 (7.0)	0 (0)	
Primary	138 (80.7)	69 (78.4)	
Secondary and tertiary	20 (11.7)	19 (21.6)	
Not known	1 (0.6)	0 (0)	
**Paternal education level, n (%)**			0.080
No School	1 (0.6)	0 (0)	
Primary	115 (67.3)	47 (53.4)	
Secondary and tertiary	51 (29.8)	40 (45.5)	
Not known	4 (2.3)	1 (1.1)	
**Maternal employment n (%)**			0.153
Yes	20 (11.7)	16 (18.2)	
No	151 (88.3)	72 (81.8)	
**Paternal employment n (%)**			0.039*
Yes	74 (43.3)	50 (56.8)	
No	97 (56.7)	38 (43.2)	
**Number of siblings n (%)**			0.380
None	33 (19.3)	19 (21.6)	
1–3	103 (60.2)	46 (52.3)	
4–6	33 (19.3)	23 (26.1)	
>7	2 (1.2)	0 (0)	
**Marital status of caregivers n (%)**			0.952
Single	5 (2.9)	2 (2.3)	
Married	154 (89.5)	79 (89.8)	
Separated	13 (7.6)	7 (8.0)	
**Maternal age in yrs. n (%)**			0.265
15–24	73 (42.6)	36 (40.9)	
25–29	49 (28.7)	18 (20.5)	
30–39	42 (24.6)	31 (35.2)	
>40	7 (4.1)	3 (3.4)	
**Paternal age in yrs. n (%)**			0.165
15–24	25 (14.6)	10 (11.3)	
25–29	54 (31.6)	18 (20.5)	
30–39	67 (39.2)	44 (50.0)	

(*Continued*)

Table 1. (Continued)

Characteristic	Severe Anaemia (n = 171)	Community Children (n = 88)	P value
>40	25 (14.6)	16 (18.2)	

Note

# The nutrition status indicators have varying total number of children assessed WAZ (n = 249); HAZ (n = 211); WHZ (n = 218) as some children did not have a score on a particular measure (height or weight) or was not corresponding to appropriate age when compared with the WHO Child Growth Standards.

* *P* value ≤ 0.05.

https://doi.org/10.1371/journal.pone.0240694.t001

deficits in domains sensitive to CNS iron levels leading to neurodevelopmental impairment [50]. Additionally, low haemoglobin concentrations adversely affect cognitive and motor development which are important risk factors for the health and development among children [51].

Our study found that children with SA were at risk of poor overall language performance specifically in expressive communication implying that children with SA may have limited ability to vocalise and perform tasks such as object naming, babbling, and gesturing strongly associated with children's overall learning, academic skills and later development [52]. Language has been shown to facilitate learning and profiling a child's skills in other domains and prediction of a child's later socioemotional, pre-linguistic and comprehension skills [52]. These early language deficits in pre-schoolers with SA may affect their cognitive and motor abilities.

Our study findings agree with Santos, Rates [53] showing that childhood anaemia has the propensity to lead to language development alterations thereby affecting social and learning abilities among pre-schoolers. Furthermore, expressive communication deficits could account for deficits in cognitive development where early child language development ability has strong associations with cognitive functioning, verbal and nonverbal skills, later development and academic achievement at school age [25, 54]. This may also affect the way in which the pre-schoolers attain language, use self-expressions when with peers of the same age and affects self-esteem and confidence.

Severe anaemia remains one of the significant public health problems among preschool children worldwide particularly in malaria prone regions and the alleviation of this condition has the potential to improve both the health of children and their neurodevelopment [23, 55, 56]. Identifying abilities that were affected provides information on how SA affects

Table 2. Frequency of neurodevelopmental deficits in children with SA compared to CCs.

	Prevalence% (95% CI)	Severe anaemia n = 171 n, (%)
**Cognition**	2.3% (0.8–6.1)	4, (2.3)
Receptive communication	1.2% (0.3–4.6)	2, (1.2)
Expressive communication	3.5% (1.6–7.6)	6, (3.5)
**Overall language**	1.7% (0.6–5.3)	3, (1.8)
Fine motor	4.7% (2.3–9.1)	8, (4.7)
Gross motor	2.3% (0.8–6.1)	4, (2.3)
**Overall motor**	3.5% (1.6–7.6)	6, (3.5)
**≥ 1 impairment#**	4.6% (2.3–9.1)	8, (4.7)

Note:

# Impairment in more than 1 area (cognition, language and motor).

* Fisher's exact test.

https://doi.org/10.1371/journal.pone.0240694.t002

Table 3. Neurodevelopmental outcome subscale z-scores in children with severe anaemia compared with Community control children.

	Unadjusted means			Adjusted means		
	**Severe anaemia n = 171 Mean, (SE)**	**Control n = 88 Mean (SE)**	**Holm's corrected p-value**	**Severe Anaemia n = 171 Mean, (SE)**	**Control n = 88 Mean (SE)**	**Holm's corrected p-value**
**Cognition**	-0.19, (0.00)	0.00, (0.00)	**0.01**	-0.20, (0.01)	0.00, (0.01)	**0.02**
Receptive communication	-0.14, (0.00)	0.00, (0.00)	0.06	-0.15, (0.01)	0.00, (0.01)	0.07
Expressive communication	-0.31, (0.00)	0.00, (0.00)	<**0.001**	-0.32, (0.01)	0.00, (0.12)	<**0.001**
**Overall language** [#]	-0.24, (0.00)	0.00, (0.00)	**0.01**	-0.25, (0.01)	0.00, (0.01)	<**0.001**
Fine motor	-0.09, (0.00)	0.00, (0.00)	0.43	-0.08, (0.01)	0.00, (0.02)	0.29
Gross motor	-0.24, (0.00)	0.00, (0.00)	**0.01**	-0.25, (0.01)	0.00, (0.01)	**0.02**
**Overall motor** [*]	-0.17, (0.00)	0.00, (0.00)	0.06	-0.17, (0.01)	0.00, (0.01)	**0.05**

Note

[#] Overall language z-score is a combined score of receptive communication and expressive communication scores.

[*] Overall motor z-score is a combined score of fine motor and gross motor scores.

Age- and sex-adjusted z-scores were computed using CC children as the reference population.

Mean z-scores were adjusted for nutrition status (weight-for-age), maternal education, paternal employment and socioeconomic status.

All p-values have been adjusted for multiple testing using Holm's procedure [Holm. S. (1979)].

https://doi.org/10.1371/journal.pone.0240694.t003

neurodevelopment and the domains that need to be evaluated post SA especially in children under 5 years. Therefore, understanding neurodevelopmental outcomes among children with SA may provide more insight on the nature and types of policies needed to address these potential child health and developmental challenges. The present study suggests that effects of SA are seen early in pre-schoolers below 5yrs of age, as lower adjusted z-scores were seen 14 days post-discharge. From our findings, SA among children below 5 years is associated with poor neurodevelopmental performance even in the context of decreasing malaria prevalence. Therefore, designing interventions that include neurodevelopmental assessment, especially in areas with high SA prevalence, could be of added value. The study results may provide direction for intervention planning and support strategies. Correction of SA among children under 5 years will enhance better neurodevelopmental outcomes supporting school performance and contributing to better health, quality of life and economic outcomes later in life [51]. Cognitive, behavioural, social and educational problems in later life could be alleviated when early identification of neurodevelopmental impairment and those at greater risk and appropriate interventions implemented among this population [51].

Our study examined neurodevelopmental performance among pre-schoolers surviving severe anaemia in Uganda using Bayley III as the primary neurodevelopmental outcome measure, which is a fairly recent neurodevelopmental performance assessment tool used worldwide and in our settings [40, 41]. However, it still needs to be standardised and culturally adapted for low resource settings like Uganda.

Certain limitations should be considered in the interpretation of the findings of the present study. It was impossible to collect and compare neurodevelopmental scores of the SA children studied before the illness. Therefore we compared children with SA to age-matched CCs to cater for other factors i.e. socioeconomic status that may interfere with the neurodevelopmental outcomes [34]. Bayley-III has not been standardized and validated in Uganda, however we converted the raw scores into z-scores for each age group based on scores of CCs to make the assessment appropriate for this study population. We lacked Hb assessment for the CC group as at the time of the study it was only considered for the severely febrile and admitted children

due to limited resources at the hospital. We also lack data related to our study sample on the underlying cause of severe anaemia common among African pre-schoolers i.e. helminth, iron deficiency, malnutrition, severe malaria, [49, 51] which could have interfered with our findings as they are potential for neurodevelopmental decline in children. Further study is required to determine whether the cognitive differences seen are due to severe anaemia or to factors such as iron deficiency or malaria that may lead to severe anaemia and also to cognitive impairment. We suggest conducting a longitudinal study following up the children to their school age years.

Conclusion

This study demonstrates that severe anaemia is associated with neurocognitive (cognition, language and motor) deficits among infants and pre-schoolers. The poor performance can be observed from as early as 6 months of age. The study findings suggest the importance of early screening and detection of neurodevelopmental deficits in pre-schoolers post illness and admission which can facilitate timely interventions for these children and others with other related conditions i.e. iron deficiency, severe malaria, and malnutrition. We propose further more rigorous investigations to examine the effect of severe anaemia in relation to long-term neurodevelopmental outcomes, as the children get older.

Supporting information

S1 File. Final Z scores by regression for neurodevelopmental outcomes data. File containing primary data used in the analysis of cognitive, language and motor outcomes.
(XLS)

Acknowledgments

We thank the children and caregivers who participated in the study; the research team (Isaac Oruru, Joan Apili, and Norine Apio) and the Lira Regional Referral Hospital administration for permission to conduct the study.

Author Contributions

Conceptualization: Andrew S. Ssemata, Robert O. Opoka, Noeline Nakasujja, Chandy C. John, Paul Bangirana.

Data curation: Andrew S. Ssemata, Robert O. Opoka, John M. Ssenkusu, Noeline Nakasujja.

Formal analysis: Andrew S. Ssemata, John M. Ssenkusu.

Funding acquisition: Chandy C. John.

Investigation: Andrew S. Ssemata, Robert O. Opoka, Paul Bangirana.

Methodology: Andrew S. Ssemata, Robert O. Opoka, John M. Ssenkusu, Noeline Nakasujja, Paul Bangirana.

Project administration: Andrew S. Ssemata, Robert O. Opoka, Noeline Nakasujja, Chandy C. John, Paul Bangirana.

Resources: Andrew S. Ssemata, Robert O. Opoka, Chandy C. John, Paul Bangirana.

Supervision: Robert O. Opoka, John M. Ssenkusu, Noeline Nakasujja, Chandy C. John, Paul Bangirana.

Writing – original draft: Andrew S. Ssemata.

Writing – review & editing: Andrew S. Ssemata, Robert O. Opoka, John M. Ssenkusu, Noeline Nakasujja, Chandy C. John, Paul Bangirana.

References

1. Kassebaum NJ, Jasrasaria R, Naghavi M, Wulf SK, Johns N, Lozano R, et al. A systematic analysis of global anemia burden from 1990 to 2010. Blood. 2014; 123(5):615–24. https://doi.org/10.1182/blood-2013-06-508325 PMID: 24297872
2. Phiri KS, Calis JC, Faragher B, Nkhoma E, Ng'oma K, Mangochi B, et al. Long term outcome of severe anaemia in Malawian children. PloS one. 2008; 3(8):e2903. https://doi.org/10.1371/journal.pone.0002903 PMID: 18682797
3. McLean E, Cogswell M, Egli I, Wojdyla D, De Benoist B. Worldwide prevalence of anaemia, WHO vitamin and mineral nutrition information system, 1993–2005. Public health nutrition. 2009; 12(4):444. https://doi.org/10.1017/S1368980008002401 PMID: 18498676
4. Stevens GA, Finucane MM, De-Regil LM, Paciorek CJ, Flaxman SR, Branca F, et al. Global, regional, and national trends in haemoglobin concentration and prevalence of total and severe anaemia in children and pregnant and non-pregnant women for 1995–2011: a systematic analysis of population-representative data. The Lancet Global Health. 2013; 1(1):e16–e25. https://doi.org/10.1016/S2214-109X(13)70001-9 PMID: 25103581
5. Legason ID, Atiku A, Ssenyonga R, Olupot-Olupot P, Barugahare JB. Prevalence of Anaemia and associated risk factors among children in North-Western Uganda: a cross sectional study. BMC hematology. 2017; 17(1):10. https://doi.org/10.1186/s12878-017-0081-0 PMID: 28680644
6. Magalhaes RJS, Clements AC. Mapping the risk of anaemia in preschool-age children: the contribution of malnutrition, malaria, and helminth infections in West Africa. PLoS medicine. 2011; 8(6):e1000438. https://doi.org/10.1371/journal.pmed.1000438 PMID: 21687688
7. Boivin MJ, Sikorskii A, Familiar-Lopez I, Ruiseñor-Escudero H, Muhindo M, Kapisi J, et al. Malaria illness mediated by anaemia lessens cognitive development in younger Ugandan children. Malaria journal. 2016; 15(1):210. https://doi.org/10.1186/s12936-016-1266-x PMID: 27076184
8. Price RN, Simpson JA, Nosten F, Luxemburger C, Hkirjaroen L, ter Kuile F, et al. Factors contributing to anemia after uncomplicated falciparum malaria. The American journal of tropical medicine and hygiene. 2001; 65(5):614–22. https://doi.org/10.4269/ajtmh.2001.65.614 PMID: 11716124
9. Dhabangi A, Idro R, John CC, Dzik WH, Siu GE, Opoka RO, et al. Community perceptions of paediatric severe anaemia in Uganda. PloS one. 2019; 14(1):e0209476. https://doi.org/10.1371/journal.pone.0209476 PMID: 30605461
10. Opoka RO, Ssemata AS, Oyang W, Nambuya H, John CC, Karamagi C, et al. Adherence to clinical guidelines is associated with reduced inpatient mortality among children with severe anemia in Ugandan hospitals. PloS one. 2019; 14(1):e0210982. https://doi.org/10.1371/journal.pone.0210982 PMID: 30682097
11. Kiguli S, Maitland K, George EC, Olupot-Olupot P, Opoka RO, Engoru C, et al. Anaemia and blood transfusion in African children presenting to hospital with severe febrile illness. BMC medicine. 2015; 13(1):21. https://doi.org/10.1186/s12916-014-0246-7 PMID: 25640706
12. Vincent JL, Baron J-F, Reinhart K, Gattinoni L, Thijs L, Webb A, et al. Anemia and blood transfusion in critically ill patients. Jama. 2002; 288(12):1499–507. https://doi.org/10.1001/jama.288.12.1499 PMID: 12243637
13. Bojang K, Palmer A, van Hensbroek MB, Banya W, Greenwood B. Management of severe malarial anaemia in Gambian children. Transactions of the Royal Society of Tropical Medicine and Hygiene. 1997; 91(5):557–61. https://doi.org/10.1016/s0035-9203(97)90025-0 PMID: 9463667
14. Menendez C, Kahigwa E, Hirt R, Vounatsou P, Aponte JJ, Font F, et al. Randomised placebo-controlled trial of iron supplementation and malaria chemoprophylaxis for prevention of severe anaemia and malaria in Tanzanian infants. The Lancet. 1997; 350(9081):844–50. https://doi.org/10.1016/S0140-6736(97)04229-3 PMID: 9310602
15. Smith JL, Brooker S. Impact of hookworm infection and deworming on anaemia in non-pregnant populations: a systematic review. Tropical medicine & international health. 2010; 15(7):776–95. https://doi.org/10.1111/j.1365-3156.2010.02542.x PMID: 20500563
16. Schellenberg D, Menendez C, Kahigwa E, Aponte J, Vidal J, Tanner M, et al. Intermittent treatment for malaria and anaemia control at time of routine vaccinations in Tanzanian infants: a randomised, placebo-controlled trial. The Lancet. 2001; 357(9267):1471–7. https://doi.org/10.1016/S0140-6736(00)04643-2 PMID: 11377597

17. Massaga JJ, Kitua AY, Lemnge MM, Akida JA, Malle LN, Rønn AM, et al. Effect of intermittent treatment with amodiaquine on anaemia and malarial fevers in infants in Tanzania: a randomised placebo-controlled trial. The Lancet. 2003; 361(9372):1853–60. https://doi.org/10.1016/s0140-6736(03)13504-0 PMID: 12788572

18. Zonnenberg IA, Vermeulen RJ, Rohaan MW, van Weissenbruch MM, Groenendaal F, de Vries LS. Severe neonatal anaemia, MRI findings and neurodevelopmental outcome. Neonatology. 2016; 109 (4):282–8. https://doi.org/10.1159/000443320 PMID: 26886231

19. Okereafor A, Allsop J, Counsell SJ, Fitzpatrick J, Azzopardi D, Rutherford MA, et al. Patterns of brain injury in neonates exposed to perinatal sentinel events. Pediatrics. 2008; 121(5):906–14. https://doi.org/10.1542/peds.2007-0770 PMID: 18450893

20. Warner-Rogers J. Clinical Neuropsychological Assessment of Children. Clinical Neuropsychology: A Practical Guide to Assessment and Management for Clinicians. 2013:317–46.

21. Sabanathan S, Wills B, Gladstone M. Child development assessment tools in low-income and middle-income countries: how can we use them more appropriately? Archives of disease in childhood. 2015; 100(5):482–8. https://doi.org/10.1136/archdischild-2014-308114 PMID: 25825411

22. Ruiseñor-Escudero H, Familiar I, Nyakato M, Kutessa A, Namukooli J, Ssesanga T, et al. Building capacity in neurodevelopment assessment of children in sub-Saharan Africa: A quality assurance model to implement standardized neurodevelopment testing. Child Neuropsychology. 2019; 25(4):466–81. https://doi.org/10.1080/09297049.2018.1497588 PMID: 30105934

23. Bangirana P, Opoka RO, Boivin MJ, Idro R, Hodges JS, Romero RA, et al. Severe malarial anemia is associated with long-term neurocognitive impairment. Clinical Infectious Diseases. 2014; 59(3):336–44. https://doi.org/10.1093/cid/ciu293 PMID: 24771329

24. Weiskopf RB, Feiner J, Hopf HW, Viele MK, Watson JJ, Kramer JH, et al. Oxygen reverses deficits of cognitive function and memory and increased heart rate induced by acute severe isovolemic anemia. Anesthesiology: The Journal of the American Society of Anesthesiologists. 2002; 96(4):871–7. https://doi.org/10.1097/00000542-200204000-00014 PMID: 11964594

25. Weiskopf RB, Kramer JH, Viele M, Neumann M, Feiner JR, Watson JJ, et al. Acute severe isovolemic anemia impairs cognitive function and memory in humans. Anesthesiology: The Journal of the American Society of Anesthesiologists. 2000; 92(6):1646–52. https://doi.org/10.1097/00000542-200006000-00023 PMID: 10839915

26. Fox SE, Levitt P, Nelson CA III. How the timing and quality of early experiences influence the development of brain architecture. Child development. 2010; 81(1):28–40. https://doi.org/10.1111/j.1467-8624.2009.01380.x PMID: 20331653

27. Wachs TD, Georgieff M, Cusick S, McEwen BS. Issues in the timing of integrated early interventions: contributions from nutrition, neuroscience, and psychological research. Annals of the New York Academy of Sciences. 2014; 1308(1):89–106.

28. Opoka RO, Ssemata AS, Oyang W, Nambuya H, John CC, Tumwine JK, et al. High rate of inappropriate blood transfusions in the management of children with severe anemia in Ugandan hospitals. BMC health services research. 2018; 18(1):566. https://doi.org/10.1186/s12913-018-3382-5 PMID: 30021576

29. Idro R, Aloyo J, Mayende L, Bitarakwate E, John C, Kivumbi G. Severe malaria in children in areas with low, moderate and high transmission intensity in Uganda. Tropical Medicine & International Health. 2006; 11(1):115–24. https://doi.org/10.1111/j.1365-3156.2005.01518.x PMID: 16398762

30. Noubiap JJ, Mengnjo MK, Nicastro N, Kamtchum-Tatuene J. Neurologic complications of sickle cell disease in Africa: a systematic review and meta-analysis. Neurology. 2017; 89(14):1516–24. https://doi.org/10.1212/WNL.0000000000004537 PMID: 28864674

31. Ministry of Health Uganda. Uganda clinical guidelines. Kampala: Ministry of Health; 2012.

32. Ballot DE, Ramdin T, Rakotsoane D, Agaba F, Davies VA, Chirwa T, et al. Use of the bayley scales of infant and toddler development, to assess developmental outcome in infants and young children in an urban setting in South Africa. International Scholarly Research Notices. 2017; 2017.

33. John CC, Bangirana P, Byarugaba J, Opoka RO, Idro R, Jurek AM, et al. Cerebral malaria in children is associated with long-term cognitive impairment. Pediatrics. 2008; 122(1):e92–e9. https://doi.org/10.1542/peds.2007-3709 PMID: 18541616

34. Bangirana P, John CC, Idro R, Opoka RO, Byarugaba J, Jurek AM, et al. Socioeconomic predictors of cognition in Ugandan children: implications for community interventions. PLoS One. 2009; 4(11):e7898. https://doi.org/10.1371/journal.pone.0007898 PMID: 19936066

35. Vyas S, Kumaranayake L. Constructing socio-economic status indices: how to use principal components analysis. Health policy and planning. 2006; 21(6):459–68. https://doi.org/10.1093/heapol/czl029 PMID: 17030551

36. Filmer D, Pritchett LH. Estimating wealth effects without expenditure data—or tears: an application to educational enrollments in states of India. Demography. 2001; 38(1):115–32. https://doi.org/10.1353/dem.2001.0003 PMID: 11227840

37. De Onis M, Blössner M. The World Health Organization global database on child growth and malnutrition: methodology and applications. International journal of epidemiology. 2003; 32(4):518–26. https://doi.org/10.1093/ije/dyg099 PMID: 12913022

38. World Health Organization. WHO child growth standards: length/height for age, weight-for-age, weight-for-length, weight-for-height and body mass index-for-age, methods and development: World Health Organization; 2006.

39. Bayley N. Bayley scales of infant and toddler development. Third Edition. 3rd ed. San Antonio, TX: Harcourt Assessment, Inc.; 2006.

40. Muhoozi GK, Atukunda P, Diep LM, Mwadime R, Kaaya AN, Skaare AB, et al. Nutrition, hygiene, and stimulation education to improve growth, cognitive, language, and motor development among infants in Uganda: A cluster-randomized trial. Maternal & child nutrition. 2018; 14(2):e12527.

41. Muhoozi GK, Atukunda P, Mwadime R, Iversen PO, Westerberg AC. Nutritional and developmental status among 6-to 8-month-old children in southwestern Uganda: a cross-sectional study. Food & nutrition research. 2016; 60(1):30270. https://doi.org/10.3402/fnr.v60.30270 PMID: 27238555

42. Singla DR, Kumbakumba E, Aboud FE. Effects of a parenting intervention to address maternal psychological wellbeing and child development and growth in rural Uganda: a community-based, cluster-randomised trial. The Lancet Global Health. 2015; 3(8):e458–e69. https://doi.org/10.1016/S2214-109X(15)00099-6 PMID: 26144389

43. Boivin MJ, Bangirana P, Byarugaba J, Opoka RO, Idro R, Jurek AM, et al. Cognitive impairment after cerebral malaria in children: a prospective study. Pediatrics. 2007; 119(2):e360–e6. https://doi.org/10.1542/peds.2006-2027 PMID: 17224457

44. Holm S. A simple sequentially rejective multiple test procedure. Scandinavian journal of statistics. 1979:65–70.

45. Hare GM. Anaemia and the brain. Current Opinion in Anesthesiology. 2004; 17(5):363–9. https://doi.org/10.1097/00001503-200410000-00003 PMID: 17023891

46. Georgieff MK. Long-term brain and behavioral consequences of early iron deficiency. Nutrition reviews. 2011; 69(s1). https://doi.org/10.1111/j.1753-4887.2011.00432.x PMID: 22043882

47. Lozoff B, Georgieff MK, editors. Iron deficiency and brain development. Seminars in pediatric neurology; 2006: Elsevier.

48. Beard J. Iron Deficiency Alters Brain Development and Functioning, 2. The Journal of nutrition. 2003; 133(5):1468S–72S.

49. Calis JC, Phiri KS, Faragher EB, Brabin BJ, Bates I, Cuevas LE, et al. Research Article (New England Journal of Medicine) Severe anemia in Malawian children. Malawi Medical Journal. 2016; 28(3):99–107. PMID: 27895843

50. Beard JL. Iron biology in immune function, muscle metabolism and neuronal functioning. The Journal of nutrition. 2001; 131(2):568S–80S. https://doi.org/10.1093/jn/131.2.568S PMID: 11160590

51. Balarajan Y, Ramakrishnan U, Özaltin E, Shankar AH, Subramanian S. Anaemia in low-income and middle-income countries. The Lancet. 2012; 378(9809):2123–35.

52. Crais RE. The Bayley-III Language Scale. In: Weiss LG, Oakland T, Aylward GP, editors. Bayley-III clinical use and interpretation. London: Academic Press, Elsevier Inc.; 2010. p. 47–75. https://doi.org/10.1044/0161-1461(2010/09-0061) PMID: 20679409

53. Santos JN, Rates SPM, Lemos SMA, Lamounier JA. Consequences of anemia on language development of children from a public day care center. Revista Paulista de Pediatria. 2009; 27(1):67–73.

54. Hohm E, Jennen-Steinmetz C, Schmidt MH, Laucht M. Language development at ten months. European child & adolescent psychiatry. 2007; 16(3):149–56. https://doi.org/10.1007/s00787-006-0567-y PMID: 17347783

55. Crawley J. Reducing the burden of anemia in infants and young children in malaria-endemic countries of Africa: from evidence to action. The American journal of tropical medicine and hygiene. 2004; 71(2_suppl):25–34. PMID: 15331816

56. Walker SP, Wachs TD, Gardner JM, Lozoff B, Wasserman GA, Pollitt E, et al. Child development: risk factors for adverse outcomes in developing countries. The lancet. 2007; 369(9556):145–57. https://doi.org/10.1016/S0140-6736(07)60076-2 PMID: 17223478
